# Supplementary material for: Identification of photoperiod-induced specific miRNAs in the adrenal glands of Sunite sheep (Ovis aries)
Source: Front Vet Sci. 2022 Jul 22;9:888207. doi: 10.3389/fvets.2022.888207 (PMC9354845; doi:10.3389/fvets.2022.888207)
Supplement: Supplementary file 1 [file Data_Sheet_1.ZIP › Supplementary file/miRDeep2 file.zip!/miRDeep2 file/Novel_Structure/LP42A3/Novel_83.pdf]

Provisional ID	:	NC_019468.2_12711
Score total	:	1.4
Score for star read(s)	:	-1.3
Score for read counts	:	0
Score for mfe	:	1.1
Score for randfold	:	1.6
Score for cons. seed	:	
Total read count	:	135
Mature read count	:	132
Loop read count	:	0
Star read count	:	3

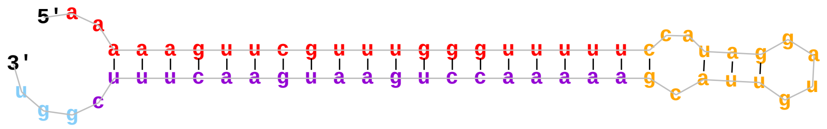

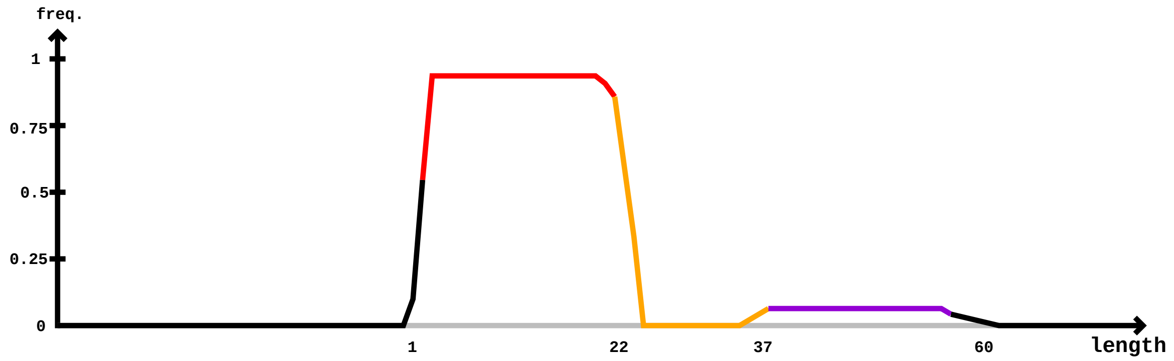

Mature Star

```
5'- gaaaccauaguaauuagaaauguuacuggcuuggccaaaaaguucguuuggguuuuuccauaggauguuacgaaaaaccugaaugaacuuucgguucaacccagaagaaug -3'  obs
    gaaaccauaguaauuagaaauguuacuggcuuggccaaaaaguucguuuggguuuuuccauaggauguuacgaaaaaccugaaugaacuuucgguucaacccagaagaaug       exp
    .........................((((.((((((..((((((((((((((((((((..(((....))).)))))))))))))))))))).)).)))).)))).......   reads   mm   sample
    ...................................caaaaaguucguuuggguuu........................................................   1   0   seq
    ...................................caaaaaguucguuuggguuuu.......................................................   4   0   seq
    ...................................caaaaaguucguuuggguuuuu......................................................   8   0   seq
    ...................................caaaaaguucguuCggguuuuu......................................................   1   1   seq
    ....................................aaaaaguucguuuggguuu........................................................   3   0   seq
    ....................................aaaaaguucguuCggguuu........................................................   1   1   seq
    ....................................aaaaaguucguuugUguuuuu......................................................   1   1   seq
    ....................................aaaaaguucguuuggguuuuu......................................................   52  0   seq
    ....................................aaaaaguucguuuggguuuCuc.....................................................   1   1   seq
    ....................................aaaaaguucguuuggguuCuuc.....................................................   1   1   seq
    ....................................aaaaaguucguuuggguuuuucA....................................................   4   1   seq
    .....................................aaaaguucguuuggguuuC.......................................................   2   1   seq
    .....................................aaaaguucguuuggguuuuuU.....................................................   10  1   seq
    .....................................aaaaguucguuuggguuuuucA....................................................   41  1   seq
    .....................................aaaaguucguuuggguuuuucG....................................................   2   1   seq
    ......................................................................Agaaaaaccugaaugaacuuu....................   1   1   seq
    ......................................................................Agaaaaaccugaaugaacuuuc...................   2   1   seq
    .......................................................................gaaaaaccugaaugaacuuu....................   1   0   seq
    .......................................................................gaaaaaccugaaugaacuuuc...................   2   0   seq
    ........................................................................aaaaaccugaaCgaacuuu....................   1   1   seq
    ........................................................................aaaaaUcugaaugaacuuuc...................   2   1   seq
```
